# Hemophagocytic lymphohistiocytosis in COVID-19: Case reports of a stepwise approach

**DOI:** 10.1097/MD.0000000000025170

**Published:** 2021-03-26

**Authors:** Sebastian Schnaubelt, Daniel Tihanyi, Robert Strassl, Ralf Schmidt, Sonja Anders, Anton N. Laggner, Hermine Agis, Hans Domanovits

**Affiliations:** a Department of Emergency Medicine, Medical University of Vienna; b Department of Pulmonology, Clinic Penzing, Vienna Healthcare Group; c Division of Clinical Virology, Department of Laboratory Medicine, Medical University of Vienna; d Department of Internal Medicine I, Medical University of Vienna, Austria.

**Keywords:** coronavirus disease 2019, hemophagocytic lymphohistiocytosis, macrophage activation syndrome

## Abstract

**Rationale:**

The immunologic syndrome induced by severe acute coronavirus disease 2019 (COVID-19) is yet not fully understood. Typical patterns of clinical and laboratory features match secondary hemophagocytic lymphohistiocytosis (sHLH). However, the optimal approach to COVID-19 patients testing positive for sHLH is still unclear.

**Patient concerns:**

Three patients with COVID-19 are reviewed. All showed hyperinflammation and cytokine storm, necessitating intensive care treatment including mechanical ventilation.

**Diagnosis:**

Secondary hemophagocytic lymphohistiocytosis due to severe COVID-19; diagnosed via HScore.

**Interventions:**

A treatment regimen of methylprednisolone, pentaglobin, and anakinra was developed and administered.

**Outcomes:**

One patient survived the ICU stay. Two other patients, in whom sHLH was diagnosed too late, deceased.

**Lessons:**

A routine screening of COVID-19 patients for secondary HLH by using the HScore is feasible; especially those patients deteriorating clinically with no sufficient response to shock management might be at particular high risk. A stepwise therapeutic approach comprising corticosteroids, immunoglobulins and anakinra, accompanied by immunoadsorption, may dampen cytokine storm effects, and potentially reduce mortality.

## Abstract 2

Supplemental Digital Content is available in the text

**Key Points**

- Secondary hemophagocytic lymphohistiocytosis may be highly prevalent in critically-ill COVID-19 patients not responding to shock management.
- A stepwise treatment regimen of corticosteroids, immunoglobulins, anakinra, and immunoadsorption may dampen cytokine storm effects, and potentially reduce mortality.

## 1 Introduction

A sepsis-like clinical picture has repeatedly been reported in coronavirus disease 2019 (COVID-19), creating a yet not fully understood syndrome. Hyperinflammation, cytokine storm, and secondary hemophagocytic lymphohistiocytosis (sHLH) are discussed as aggravating factors.^[1,2]^ Both sHLH mortality rates in non-COVID-19 patients (around 40%) and in critically-ill COVID-19 patients (around 65%) are high, and viral infections are known as sHLH triggers.^[1,2]^ Immunosuppression has been suggested as a treatment option,^[3,4]^ and first reports are promising.^[1]^ In this context, more data on clinical management of sHLH triggered by COVID-19 are urgently expected

## 2 Methods

### 2.1 Patients

We reviewed COVID-19 patients admitted to an intensive care unit in Vienna, Austria between April and May 2020, who were diagnosed with sHLH. Patients’ clinical-, imaging-, and laboratory data (see Supplements, http://links.lww.com/MD/F969 and the supplemental figure, http://links.lww.com/MD/F968) were assessed.

### 2.2 SARS-CoV-2 diagnosis

Testing for the presence of severe acute respiratory syndrome coronavirus 2 RNA in pharyngeal or tracheal respiratory specimens was performed by Real-Time qPCR. Positive results (Ct value >35) were confirmed by repeated testing.

### 2.3 sHLH diagnosis

sHLH was diagnosed using the HScore:^[5]^ Nine variables are assessed: core temperature, hepato- and/or splenomegaly, number of cytopenias, levels of TG, fibrinogen, ferritin and ASAT, history of immunosuppression, and (if feasible) presence of bone marrow haemophagocytosis. A positive result yields a 93% sensitivity and 86% specificity for HLH.

### 2.4 Immunosuppressive therapy

Immunosuppressive treatment for sHLH was conducted in a stepwise approach:

1. 1 g of methylprednisolone intravenously once daily for 3 days,
2. 1 g/kg of Pentaglobin (50 mg/ml human plasma protein containing ≥95% of immunoglobulin [6 mg IgM, 6 mg IgA, 38 mg IgG], Biotest Corp., Dreieich, Germany) via continuous infusion over 48 hours,
3. 200 mg of anakinra subcutaneously twice daily until clinical improvement. Anakinra was used as an off-label salvage treatment.

### 2.5 Patient consent and ethical review

All data have been anonymized. Informed consent for publication of anonymized data from the patient or their relatives have been obtained. Ethical review was not necessary for case reports following local respective guidelines.

### 2.6 Patient 1

A 51-year old male (BMI 26.2) with a fever for 6 days was hospitalized due to respiratory failure and tested positive for SARS-CoV-2. Showing acute respiratory distress syndrome (ARDS), he was intubated and mechanically ventilated. Acute kidney injury (AKI) necessitated continuous renal replacement therapy (CRRT), upgraded with an immunoadsorption filter (day 5 of hospitalization) against cytokine storm. Despite noradrenaline support, the hemodynamic profile deteriorated. Dobutamine was added due to heart failure with reduced ejection fraction and impaired left ventricular function. While levosimendan, argipressin, and landiolol led to a transient clinical improvement, hemodynamics further worsened. On the 21st day of hospitalization, sHLH was diagnosed (Table 1), and immunosuppressive therapy was started with methylprednisolone for 72 hours, followed by Pentaglobin (see *Methods*). After 26 days of ICU treatment, hemodynamics further deteriorated, and he deceased due to multi organ failure.

**Table 1:** Patients’ initial sHLH diagnosis details including Hscore (5) points and the subsequent course of sHLH from the day of diagnosis onwards, monitored through the Hscore.

	Patient 1			Patient 2			Patient 3		
Initial diagnosis	Parameter	Value	Hscore points	Parameter	Value	Hscore points	Parameter	Value	Hscore points
	temperature (°C)	38.2	33	temperature (°C)	37.0	0	temperature (°C)	39	33
	organomegaly	liver & spleen	38	organomegaly	liver	23	organomegaly	liver	23
	number of cytopenias	2	24	number of cytopenias	2	24	number of cytopenias	2	24
	triglycerides (mmol/L)	10.5	64	triglycerides (mmol/L)	6.08	64	triglycerides (mmol/L)	4.9	64
	fibrinogen (g/L)	4.97	0	fibrinogen (g/L)	9.95	0	fibrinogen (g/L)	7.03	0
	haemophagocytosis in bone marrow aspirate	n.a.	0	haemophagocytosis in bone marrow aspirate	n.a.	0	haemophagocytosis in bone marrow aspirate	n.a.	0
	ferritin (μg/L)	9858	50	ferritin (μg/L)	7377	50	ferritin (μg/L)	3558	35
	serum ASAT (IU/L)	108	19	serum ASAT (IU/L)	173	19	serum ASAT (IU/L)	82	19
	known immunosuppression	no	0	known immunosuppression	no	0	known immunosuppression	no	0

course of sHLH	day of hospitalization	Hscore points	positive for sHLH	day of hospitalization	Hscore points	positive for sHLH	day of hospitalization	Hscore points	positive for sHLH
	21 (= day of sHLH diagnosis) to 23	228	yes	19 (= day of sHLH diagnosis)	180	yes	16 (= day of sHLH diagnosis)	198	yes
	24	195	yes	20	156	no	17 to 19	141	no
	25	180	yes	21 to 23	180	yes	20 to 22	165	no
	26	195	yes				23 to 25	141	no
							26	198	yes
							27 to 28	174	yes
							29 to 35	141	no

°C = degrees Celsius, μg = micrograms, ASAT = aspartate-aminotransferase, g = grams, IU = international units, L = liter, mmol = millimole, sHLH = secondary hemophagocytic lymphohistiocytosis.

### 2.7 Patient 2

Delirium and dyspnea for 4 days were reported by a 75-year old man (BMI 29.4) before he was hospitalized and tested positive for SARS-CoV-2. ARDS led to intubation, mechanical ventilation, and intermittent prone positioning. CRRT with immunoadsorption due to AKI and cytokine storm were established (3rd day of hospitalization). Deteriorating heart failure with reduced ejection fraction and intermittent noncompensatory tachycardia necessitated a treatment regimen of noradrenaline, dobutamine, agripressin, landiolol, and levosimendan, leading to a sustainable hemodynamic improvement. On the 19th day of hospitalization, sHLH was diagnosed (Table 1). Intravenous methylprednisolone was started, but before escalating the immunosuppressive therapy, fulminant pulmonary embolism occurred, not responding to systemic thrombolysis, and resulting in a fatal outcome on the 23rd day.

### 2.8 Patient 3

A 74-year old woman (BMI 19.4) was tested positive for SARS-CoV-2. Aggravating dyspnea and delirium – not manageable by noninvasive ventilation – necessitated intubation due to ARDS. Cytokine storm and AKI were present; CRRT with immunoadsorption was initiated on the 18th day. Mild noradrenaline support was necessary to sustain a stabile hemodynamics. sHLH was diagnosed on the 16th day of hospitalization (Table 1). Steps 1 to 3 of the described immunosuppressive regimen were administered *(see Methods)* without serious adverse events. Markers of cytokine storm and sHLH regressed, and CRRT and hemodynamic support could be stopped. After 35 days of ICU-care and negative tests for SARS-CoV-2, the patient entered a successful weaning process and was still alive at a follow-up on day 85.

## 3 Discussion

### 3.1 Complex pathophysiology

All 3 COVID-19 patients showed cytokine storm, and their clinical course is in line with typical clinical- (fever, hypoxia, delirium) and laboratory (hyperferritinemia, lymphopenia, elevated IL-6, and CRP) features of critically-ill COVID-19 patients in general,^[3,6]^ and those with severe cardiac injury or coagulopathy.^[3,7]^ A surge of inflammatory cytokines, ensuing hyperinflammation and tissue damage, is suspected as the main mechanism for multiorgan failure.^[2,3,7]^ This suggests a considerable percentage of critically-ill COVID-19 patients suffering from sHLH, which – if left undiagnosed – could explain high mortality rates.^[3,4]^ COVID-19 cytokine profiles resemble those in sHLH, strengthening this theory.^[8]^

### 3.2 An under-diagnosed syndrome

sHLH is often unrecognized and remains a diagnostic challenge. Several scoring systems have been developed.^[2,9]^ The Hscore postulated by Fardet et al^[5]^ makes a quick evaluation possible. So far, no randomized-controlled trials for sHLH treatment regimens are available, and data remains scarce even in the non-COVID-19 population. Immunomodulation has shown the potency to reduce mortality and was also suggested in COVID-19.^[4]^ Cytokine storm may respond well to immunosuppressive agents such as tocilizumab (IL-6 receptor antibody) or anakinra (IL-1 receptor antagonist). Moreover, reports show a benefit of combining corticosteroids and immunoglobulins in severely-ill COVID-19 patients.^[1,3,4,7,10]^

### 3.3 A stepwise treatment-approach

**Figure 1:**
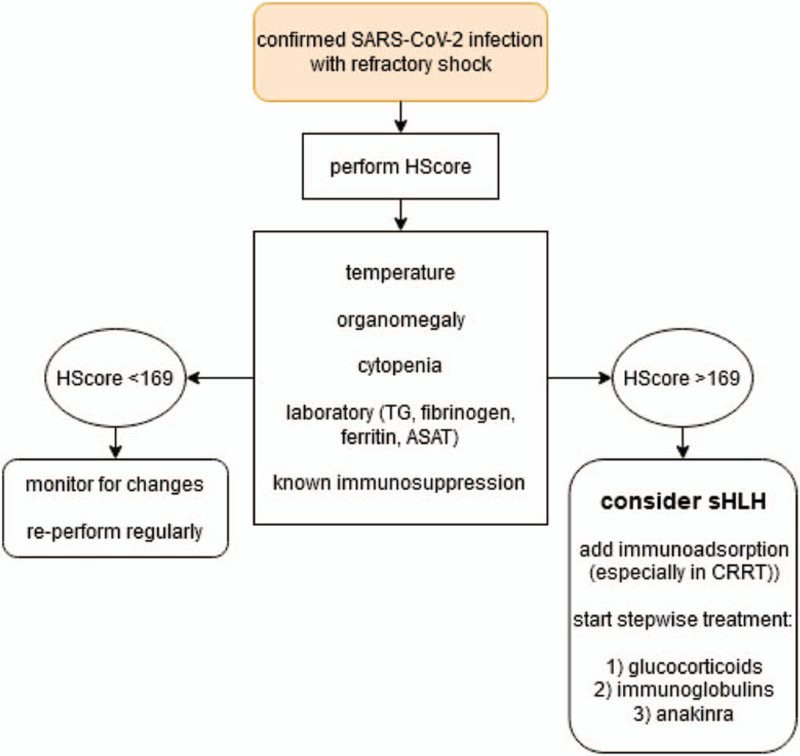
Flowchart for the suggested diagnostic and therapeutic approach concerning secondary haemophagocytic lymphohistiocytosis (sHLH) in COVID-19 patients with refractory shock. The Hscore as published by Fardet et al^[5]^ is used. ASAT = aspartate-aminotransferase, CRRT = continuous renal replacement therapy, SARS-CoV-2 = severe acute respiratory syndrome coronavirus 2, TG = triglycerides.

All our patients showed the full sHLH characteristics.^[2,4,5]^ As discussed before,^[2]^ we applied and suggest a three-step approach to sHLH caused by COVID-19 (see Fig. 1 and *Methods*): initial immune attenuation through high-dose pulsed methylprednisolone, followed by a bodyweight-adapted dose of immunoglobulins, and lastly anakinra until clinical improvement. This approach bears the benefits of a cheap and easy-to-obtain substance as the initial line of attack, followed by the stronger immunomodulatory agents. In parallel, we recommend immunoadsorption for cytokine storm dampening, especially in CRRT.

We believe that sHLH diagnosis came too late to reverse outcomes for patients 1 and 2, whereas our approach led to a favourable outcome in patient 3 - similar as reported by Dimopoulus and colleagues.^[1]^

### 3.4 Early diagnosis is key

The risk of diagnosing sHLH too late must be weighed against the potential side effects of aggressive immunosuppression,^[2]^ such as coagulopathy after immunoglobulin application. However, we believe that routine sHLH-screening of COVID-19 patients and early goal-directed therapy can lead to improved outcomes. sHLH should especially be considered in patients deteriorating fast without sufficient response to shock management. As the magnitude of cytokine levels may not correlate with sHLH severity and since specific markers (e.g. soluble-IL-2-receptor) are often not available, trends in ferritin could be used in treatment response tracking and outcome prognostication.^[2,7]^ Special attention should be paid to a possible rebound after treatment discontinuation.^[2]^ First reports of sHLH treatment in COVID-19 are encouraging,^[1]^ and results of a prospective COVID-19 sHLH cohort are expected for late 2020 (Clinical Trials-ID: NCT04347460). However, further interventional trials are needed to confirm our assumptions.

## 4 Conclusion

Routine screening for sHLH in COVID-19 using the HScore appears reasonable; patients without sufficient response to shock management might be at particular risk. A stepwise therapeutic approach comprising corticosteroids, immunoglobulins, and anakinra, accompanied by immunoadsorption, may dampen cytokine storm effects and reduce mortality.

## Acknowledgments

We thank the Viennese nursing and physician staff for their continuous efforts during the ongoing pandemic.

## Author contributions

**Conceptualization:** Sebastian Schnaubelt.

**Data curation:** Sebastian Schnaubelt, Daniel Tihanyi.

**Formal analysis:** Sebastian Schnaubelt, Daniel Tihanyi, Robert Strassl, Ralf Schmidt, Hermine Agis.

**Investigation:** Sebastian Schnaubelt, Robert Strassl, Sonja Anders, Hermine Agis.

**Methodology:** Sebastian Schnaubelt, Robert Strassl, Sonja Anders, Hermine Agis.

**Project administration:** Sebastian Schnaubelt, Sonja Anders, Hans Domanovits.

**Resources:** Sebastian Schnaubelt, Daniel Tihanyi, Robert Strassl, Ralf Schmidt.

**Software:** Sebastian Schnaubelt, Robert Strassl, Ralf Schmidt.

**Supervision:** Sonja Anders, Anton N Laggner, Hans Domanovits.

**Validation:** Sebastian Schnaubelt, Daniel Tihanyi, Robert Strassl, Hermine Agis, Hans Domanovits.

**Visualization:** Sebastian Schnaubelt.

**Writing – original draft:** Sebastian Schnaubelt.

**Writing – review & editing:** Sebastian Schnaubelt, Daniel Tihanyi, Robert Strassl, Ralf Schmidt, Sonja Anders, Anton N Laggner, Hermine Agis, Hans Domanovits.
